# Modern Developments of the Voluntary Hospital System

**Published:** 1901-10-05

**Authors:** 


### MODERN DEVELOPMENTS OF THE VOLUNTARY HOSPITAL SYSTEM.

By the Author of "The Hospital Commissariat."

In a previous series of articles we attempted to trace the successive steps which were taken by the citizens of London to provide for the sick poor of the metropolis. The causes to which the varied character of the London hospital system is due were examined and the history was brought down to the introduction of antiseptic surgery in the early seventies, inaugurating an entirely new epoch in hospital development.

No one whose memory can bridge over that period of 30 years can fail to be deeply impressed with the silent revolution they have seen accomplished all around in habits and environment ; but it is in the hospital that the change is most startling, and that it has produced the most striking results.

From this point the growth of the London hospital is no longer to be tested solely by bricks and mortar. The founding of new voluntary institutions is checked. The flow of voluntary offerings for the service of the sick poor continues to increase in volume and to widen in extent, but it is taxed to the uttermost in improving, enlarging and refitting the old establishments, in providing accommodation for which former generations saw no need, and appliances superseded in rapid succession as science advances.

The growth of population in London has never ceased to constitute a strong incentive to expansion in hospital work, and the numbers requiring free medical aid have been multiplied of late years not only by the expansion of Greater London and the unprecedented increase in its inhabitants, but by the advance of medical and surgical science. Many sufferers from complaints now readily identified as suitable for hospital treatment would not many years ago have been classed vaguely as cases of " decline ;" many more would have been dismissed as incurable. The books of the past century reveal the existence in nearly every household of some chronic invalid, often the mother of the family, suffering from some ill-defined lingering complaint which confined her to the sofa for the rest of her life. The chronic invalid was no less frequent in the homes of the poor, though the harsh conditions of life might shorten the slow decay. It would hardly be too much to say that the advance of medical knowledge has doubled during the last 30 years the cases per cent. of the sick population deemed suitable for hospital treatment.

The increase in this direction has, however, been met, and to some extent equalised, by the marked diminution in the average length of each person's stay in the hospital wards. From an average of 40 or 50 days, or even longer, the modern hospital has been able to reduce the patient's stay to from 20 to 25 days, so that, whereas in former days six or eight patients would be accommodated in the course of the year in each bed, 16 or 18 can now be benefited.

That the hospitals have altogether failed, however, to keep pace with the spread of the population in London, even with the very large additions which they have in almost every instance succeeded in making to their wards, is indisputable. Had the number of general hospitals in London been doubled during the past 30 years, in which, as we have said, no important additions to their number have been made, the accommodation thus provided would still to-day be far short of the actual requirements.

The establishment and admirable equipment of the Poor Law infirmaries during this period have had a very marked influence on hospital development. They have relieved the voluntary system of what might truly be called the "intolerable strain" of attempting to house all the destitute sick of this vast city ; they have rendered careful selection possible, and they have set the hospitals free to do even higher service for the nation than was dreamed of by their pious founders.

An examination of the number of beds available for the sick poor of the metropolis in the Poor Law infirmaries, and the cost at which they are maintained, reveals in a striking manner the magnitude of the task under which, prior to their establishment, the hospitals laboured, and the extent of unrelieved misery subsisting at their very doors.

The gigantic establishments of the Metropolitan Asylums Board have also had their influence on the voluntary hospitals, nearly all of which were compelled in old days to maintain large isolation wards for the care of infectious cases inadvertently admitted, even where this branch of the work was not an avowed feature.

By these entirely new branches of public service, and by the now universal adjunct of the convalescent home, the work of the voluntary hospital in London has been stripped of much which clogged its progress in the past. The work which in early days was deemed all important of shielding from the eyes of the compassionate the sight of unrelieved suffering has been absorbed by the Poor Law authorities. The protection of the citizen from the contagion of the fever stricken has been undertaken by the city. There are homes for the dying and homes for the partially recovered, and for the lessened ranks of the incurable there are homes also.

The hospitals are now at length unhampered by any necessity for receiving in the sacred name of charity patients they are unable to cure or have no reasonable expectation of benefiting. They are able to concentrate their energies on the work which can in no rate-supported or State institution be so well performed, that of bringing to bear the highest skill, the most perfect appliances, and the most careful investigations of modern science for the benefit of those who from the nature of their disease and from their general circumstances are best fitted for treatment.

But side by side with the great work of healing carried on in the voluntary hospitals have grown up other branches of public service, in no other country performed exclusively by voluntary effort.

The work of medical education has, during the past century, come to be entirely identified with hospital work. The modern institutions for the sick, inaugurated by the State and by the municipality, are rigidly closed to the student. The outside public sometimes ignorantly suppose, and are sometimes wilfully misinformed, that the presence of students in the wards of our great hospitals is a source of distress to the patients, and a cause of hardness and indifference to suffering among the young men themselves. To refute such false impressions it is only necessary to go back to earlier times before the student was admitted to hospital instruction and to read the testimony of eye-witnesses to the evils which could subsist where no free current of observation was admitted. Or, if the testimony of a former age be not admitted, let the story of the Poor Law relief of the sick be studied and some juster views of the advantages reaped by the sick poor under the union of medical education with voluntary hospital work will be gained. Perhaps no system could be devised better calculated to bring out all that is good in a man, induce gentleness, self sacrifice, and patience than that under which his medical education is carried out under the wholesome influence of direct and natural association with the suffering poor.

During the last 30 years the hospitals have undertaken an entirely new work in the training of nurses for the public service. So unobtrusively has this been done that though the term "hospital-trained nurse" has come to stand as the only description of a nurse who knows her business, few people, if any, realise the enormous sums of money which are expended year by year in the voluntary hospitals of London to provide the public with this valuable adjunct to medical and surgical skill.

Lastly, the voluntary hospitals of London are beginning, in obedience to the expressed will of many of the founders in past ages, to dedicate some portion of their energies to the investigation of the vast material gathered under their roofs. On this we will not touch further at this moment, except to observe that among all the signs of renewed zeal and tender charity shown by the directors of these great charities of late years, it is this which gives rise to the highest expectations of their permanent value.

Not long ago the writer was taken through a series of special wards in one of the general hospitals of London. Brightness and comfort were magically combined, delicately tinted walls, glowing fires, couches, books and pictures, costly flowers, and the attendance of skilled and sympathetic women, the anxious supervision of some of the best surgeons of London. All traces of suffering and disease seemed to be effaced, and even in the eyes of the patients was a look of rest. I turned at the door of the wards and looked back over the peaceful scene as the sun streamed in through the south window. "In the course of three months from now," whispered my conductor, "all these people will be dead ; we can do nothing for them except keep them from suffering very much.

And were this the last word how paralysing in its despair: yet even here a vital ray of hope is burning. Patiently, humbly, very cautiously the work of examination, investigation and tabulation goes on; not fitfully as the tired surgeon snatches occasional moments from his night's rest to record observations made under the stress of routine; but with all the strenuousness of his energies devoted to no other thing. And so in time—perhaps for another generation—may the hope be realised in the chambers of death itself the secret of life be recovered.

Who shall deny to the voluntary hospitals of London the honour of this work?

